# Carbon-Neutral Silicon via Aluminothermic Reduction? Exploring Industrial Symbiosis through Life Cycle Assessment

**DOI:** 10.1021/acssuschemeng.5c04666

**Published:** 2025-09-02

**Authors:** Elisa Pastor-Vallés, Alejandro Abadías Llamas, Johan Berg Pettersen

**Affiliations:** † Industrial Ecology Programme, Department of Energy and Process Engineering, 8018 NTNU Norwegian University of Science and Technology, Kolbjørn Hejes vei 1B, Trondheim 7034, Norway; ‡ Nordenham Metall GmbH, Nordenham 26954, Germany

**Keywords:** Si, Al dross, carbon footprint, LCA, prospective, circular economy, industrial symbiosis, sustainable metallurgy

## Abstract

Silicon is conventionally produced by carbothermic reduction, which reduces quartz with a carbon source. An alternative process is the aluminothermic reduction, which uses an aluminum source instead, leading to a substantial decrease in direct CO_2_ emissions. This paper assesses a case study on industrial symbiosis by producing silicon through aluminothermic reduction using aluminum dross resourced as a reductant material. Various process alternatives are evaluated, with inventories constructed from thermodynamic process simulations and mass and energy balances. We find that the impact of global warming and cumulative energy demand can be reduced by up to 80% in the aluminothermic route. Still, other impacts increase due to the strong influence of the expected alternative use of the aluminum scrap fraction and the need for additional input materials. From the different process parameters and configurations studied in the aluminothermic route, recirculating carbonation gases, reprocessing the byproduct slags, and the use of surplus aluminum scrap hold the most significant potential. The methodology used in this article exemplifies the use of prospective Life Cycle Assessment (LCA) in support of concept development to identify environmental hotspots and improvement potential in the early phases of production technologies.

## Graphical abstract

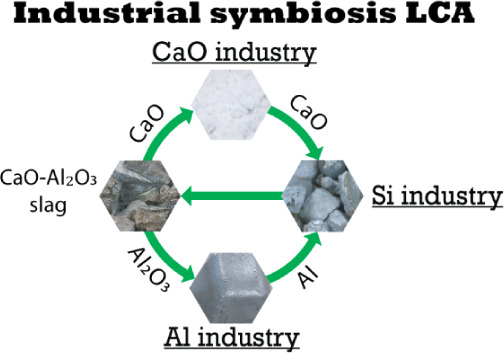

## Introduction

The transition toward a low-carbon economy will require increased production of materials such as silicon, which is key in producing photovoltaics, electronics, or aluminum alloys for applications such as lightweight components and electric vehicles. Because of their high economic importance and supply risk, both silicon and aluminum are considered Critical Raw Materials (CRMs) for the European Union.^1^ However, conventional silicon and aluminum production generate significant global warming emissions—nearly 2% of global CO_2_-eq emissions—where the most significant footprint corresponds to indirect emissions from the electricity used in these processes.^2^ While cleaner energy can reduce indirect emissions, direct emissions from the reduction with carbon are more difficult to abate.

Silicon is traditionally produced via carbothermic reduction, where quartz or silicon dioxide is reduced using a carbon source (eq 1).

1 $$\left(1+x\right){\mathrm{SiO}}_{2}+\left(2+x\right)C+\left(1+x\right)O_{2}\to \mathrm{Si}+{x\mathrm{SiO}}_{2}+\left(2+x\right){\mathrm{CO}}_{2}+\mathrm{heat}$$

where parameter *x* is related to the silicon yield and depends on the furnace operation and raw material properties.^3^

Because carbon is utilized due to its chemical properties and not its energy content,^4^ reducing CO_2_-generated emissions below this stoichiometric level is impossible without substituting the reducing agent away from carbon.

A noncarbon-based alternative for silicon production is metallothermic reduction, which replaces carbon with reactive metals. Due to their high affinity with oxygen, these metals enable reduction at lower temperatures than carbothermic processes.^5^ When aluminum is used as a reductant to produce silicon, the process is called aluminothermic reduction (eq 2).

2 $${\mathrm{SiO}}_{2}+4/3\mathrm{Al}\to \mathrm{Si}+2/3{\mathrm{Al}}_{2}O_{3}+\mathrm{heat}$$

The aluminothermic reduction process avoids direct CO_2_ emissions and offers the potential to use aluminum residues like dross. An industrial symbiosis opportunity arises by using secondary input materials and through the generation of byproducts of potential economic value. However, the fact that carbon is removed from production does not ensure carbon neutrality, when CO_2_ emissions have no net effect over the climate, as emissions may increase from a systems perspective. For instance, diverting aluminum residues from recycling, which consumes only 5% of the energy of primary production^6^, could have unintended environmental trade-offs. Raw material impurities could also affect furnace operations.

Within the Technology Readiness Level framework (TRL),^7^ the aluminothermic technology is still at TRL 6, where the technology has been demonstrated in a relevant environment close to real-world conditions, but still not fully deployed. In contrast, the carbothermic route is a TRL 9 technology, where the actual system is proven in an operational environment. Because at the earlier stages of technology development there is more freedom to modify its core characteristics, prospective Life Cycle Assessment is used to model the environmental impacts of a technology at a future state. This forward-looking approach enables the study of key parameters in the environmental impact, which can be modified before large-scale implementation.^8^ To address data gaps, secondary and proxy data are used to scale-up lab processes with generally lower yields.^9^

In this study, we evaluate the environmental performance of metallurgical-grade silicon (MG-Si, 97–99% purity) through aluminothermic reduction using prospective Life Cycle Assessment (LCA), and compare it with the carbothermic production process.

## Material and Methods

Aluminothermic reduction is still at a low maturity level (currently TRL6) compared to carbothermic production, which is fully industrialized (TRL9). To compare both production routes, we develop process simulations in HSC Chemistry^10^ to generate and validate prospective inventory data, which Parvatker and Eckelman^10^ suggested as the most accurate way to establish inventories for prospective LCAs, second to getting plant data. Simulating both routes improves comparability and establishes a benchmark for the carbothermic production. Besides a recently published parametric LCA on the conventional route,^11^ most existing Si LCA studies have focused on solar (SoG-Si) or electronic (EG-Si) applications only,^12–17^ with further refining steps from conventional metallurgical-grade silicon (MG-Si).^3,18^

The framework to develop this LCA is under the ISO 14040-14044,^19,20^ which defines the development of LCAs into four iterative phases: (i) goal and scope definition, where the methodological choices of the study are defined; (ii) inventory analysis, or the compilation and quantification of inputs and outputs to the system of study; (iii) impact assessment, which by assigning an environmental load to each flow in the inventory evaluates the environmental burdens for a system throughout its life cycle; and (iv) interpretation, in which the previous findings are evaluated according to the defined goal and scope.

### Goal and Scope Definition

This research aims to benchmark the aluminothermic route of silicon production, a potentially decarbonized alternative, and compare it to the carbothermic (conventional) route, identifying hotspots and potential areas of improvement. The functional unit (F.U.) selected for comparison is 1 tonne of metallurgical-grade silicon (MG-Si).

Methodological choices include:

- Implementation is assumed to take place in Europe under average regional conditions, aligning with the European objective to ensure a sustainable supply of CRMs. Moreover, a recent revision of the EU’s Waste Shipment Regulation (WSR), which may limit nonhazardous waste export to non-OECD countries,^21^ could increase the availability of secondary resources such as aluminum dross for aluminothermic reduction.

- Mass and energy balances are developed through HSC Chemistry models verified with lab and pilot tests. Scaling up inventories from the current pilot stage to full maturity process makes it possible to compare the incumbent and emerging Si production technologies.
- Brightway2 is used to perform the calculations through its GUI, the Activity Browser. The database used is Ecoinvent 3.9.1, Allocation at Point of Substitution (APOS).
- This study considers system expansion (substitution) to account for multifunctionalities, drawing a consequential perspective as recommended by the ILCD for meso/macro-level decision contexts.^22^ When a byproduct is produced, it replaces the production of the same material in the most likely alternative way of producing it. Similarly, when a secondary resource is consumed, the environmental load of the material that would otherwise be available for use in other processes is assigned to the consuming process.

The system adopts a cradle-to-gate approach (Figure 1) and excludes downstream impacts because both silicon products serve identical markets and further processing.

**Figure 1:**
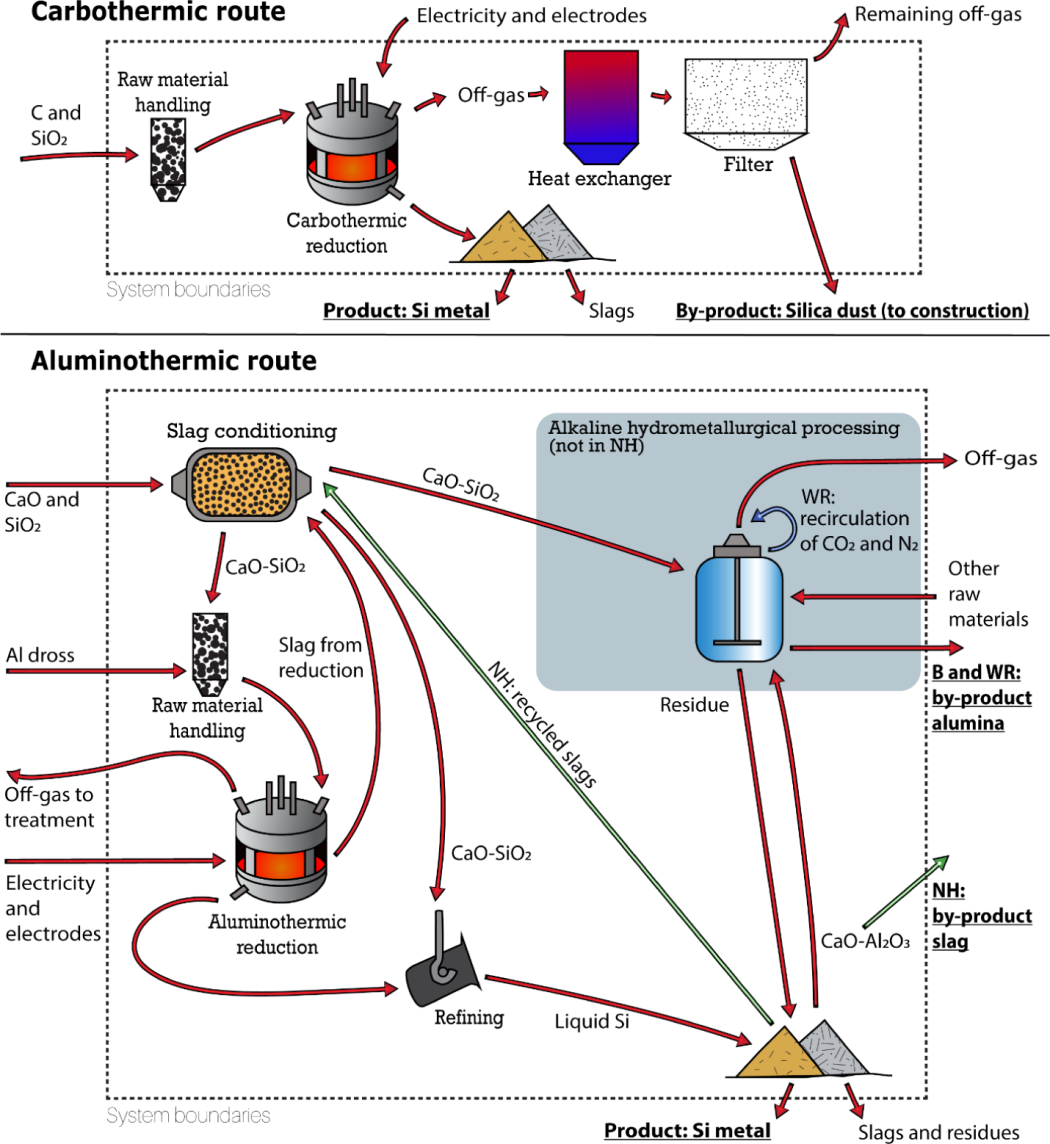
System flowcharts. Various process configurations have been analyzed in the aluminothermic route following the prospective approach. The red arrows represent the baseline process, while the blue and green arrows only include flows from the Aluminothermic with recirculation (WR) and Aluminothermic no-hydrometallurgy (NH), respectively.

Aluminothermic reduction opens several opportunities for industrial symbiosis, by using secondary input materials and generating byproducts of potential economic value. Besides, because it avoids the use of carbon, it is also free of direct CO_2_ emissions from the oxidation of this material.

We explore Al white dross as a source of aluminum and silicon sculls as a source for silicon in the aluminothermic route. White dross is high-metallic content aluminum dross from primary production, generally valorized in the aluminum and steel industries,^23^ with an aluminum metal content in the range of 15–80%.^24^ Silicon sculls (or skulls) are recovered materials from conventional Si production that remain in vessel walls after melting.^25^

The aluminothermic reduction process is divided into three steps in the baseline configuration: first, silicon dioxide contained in Si sculls is melted with calcium oxide in a furnace to generate a CaO-SiO_2_ slag—slag is defined as the byproduct generated during high-temperature pyrometallurgical processes, which floats in the surface of the molten metal and protects it from oxidation.^25–28^ The slag is then reduced with Al white dross, forming silicon and a CaO-Al_2_O_3_ slag. The reduced slag can subsequently be subject to a hydrometallurgical process to extract and recover its alumina content, a byproduct of this route, returning calcium oxide for its application in the slag-making step.

Two potential modifications of this process include:

- Aluminothermic WR (with recirculation): the carbonation gas (nitrogen and carbon dioxide) is recirculated to decrease material demand.
- Aluminothermic NH (no-hydrometallurgy): the hydrometallurgical route to obtain alumina does not take place, and the slag byproduct is valorized due to its high percentage of CaO. In addition, due to a high content of SiO_2_, silicon refining slags are also being recirculated inside the process as feed material, avoiding an extra consumption of quartz.

Industrial deployment of the aluminothermic route rests on achieving a good symbiotic setup between the silicon, aluminum, and quicklime industries. While aluminum dross and quicklime are required as reductant and for the slag conditioning and refining, respectively, both industries can also benefit from the coproducts of the aluminothermic production of silicon. Achieving a good symbiotic setup requires both access to raw materials and product markets, as well as cost-efficient logistics to minimize transport and handling losses, possibly through colocated plants. In comparison, the carbothermic route yields a single byproduct (condensed silica fume^3^), used as pozzolanic material in the concrete industry.^29^ Implementation of this exchange is more straightforward because a single industry is involved, and the market is already developed.

Regarding the availability of secondary materials for the aluminothermic route, around four million tonnes of white dross are produced annually,^30^ almost the equivalent to the global silicon production in 2024.^31^ Aluminum white dross, however, has other market segments that might compete with this use, as in comparison, the demand for aluminum ascended to 78 million tonnes the same year.^32^ While aluminum industry recycling is a competitor in the use of aluminum residues, the fact that the aluminum industry is also the biggest consumer of silicon^33^ might favor industrial symbiosis opportunities between these industries. Moreover, the market for silicon in photovoltaics is increasing rapidly, and the IEA predicts renewables will meet almost half of global electricity demand by 2030, of which solar energy will account for 80% of the capacity growth.^34^

### Inventory Analysis

Thermodynamic process simulations are model-based representations of physical, chemical, thermal or other technical processes using process simulation software. As a result, mass and energy balances are used as a source for the inventory of an LCA of metallurgical processes.^35^

Operating parameters for the aluminothermic route were defined based on experiments, chemical equilibrium calculations with FactSage, and internal data. The conventional route for producing silicon was simulated through data from industrial partners of the SisAl project and literature. Thermodynamic process simulations were developed in HSC Chemistry, and all models were validated with pilot and industrial partners. Detailed information on the models can be found in Supporting Information A.

Since thermodynamic process simulations consider a high purity of reductants and complete combustion, these models did not cover some pollutants that had to be added through emission factors. For the carbothermic production route, pollutants added include NOx and SO_2_,^36^ CH_4_,^37–39^ dioxins and PAH,^40^ and particulate materials.^41^ These are shown in Table S1.

The majority of NOx formed during the carbothermic reduction process originate in the area between the charge burden and smoke hood by the oxidation of nitrogen at high temperatures (above 1400 °C).^42–44^ In this area, the exothermic oxidation of SiO is produced in contact with air, which contributes to raising the temperature of the process. In fact, the production rate of NOx in the conventional route is proportional to the release of SiO gas,^45,46^ and temperature.^47^ Because the aluminothermic reduction takes place at lower temperatures, SiO is only created at tapping, when Si metal is exposed to air. NOx emissions from tapping are normally attributed less than 10% of NOx emissions, which will depend on tapping procedures.^42,44,48,49^

In addition, pollutants with an organic origin, such as PAH, SO_2_ and dioxins, are considered negligible since the organic materials are not fed into this production route. PM2.5 was estimated to account for less than 3 μg/m^3^ in the off-gas.^50^

Other trace metals contained in the raw material and electrodes, which can escape through the filtered off-gas, are estimated from a study on parametric LCA of silicon production.^11^ Emissions from electrodes are also included in the aluminothermic reduction, as this reaction still needs carbon electrodes for heating.

Both biocarbon and fossil carbon are used in the carbothermic route, assuming conventional raw material feed mix in Europe (around 35% biocarbon, according to industrial data). Biocarbon in the form of woodchips and charcoal has long been used for silicon production.^51,52^ We adopt the IPCC recommendation to consider biogenic CO_2_ emissions as carbon-neutral. Biogenic emissions are calculated and discounted from the total carbon emissions for the carbothermic route, as recommended by the IPCC,^53^ following the reductants’ chemical analyses from the Phyllis Classification database.^54^ These are shown in Table S2.

### Impact Assessment and Interpretation

This study is limited to four impact categories because off-gas measurements are not developed on a lab scale, and thus, inventorizing other interactions with the environment remains challenging. However, the impact categories selected are considered relevant to the circular economy, to which societal performance might change over time: climate change and water consumption (ReCiPe 2016 ^55^), abiotic depletion potential (CML-IA V4.8, material resources^56^) and Cumulative Energy Demand (total energy content).

## Results and Discussion

### Inventory Analysis

In the aluminothermic route only the aluminum metal in the dross can take part in the reduction process. Data from industrial partners places average white dross composition at 62% Al metal, 13% alumina (Al_2_O_3_), and 25% other metals and oxides. Following the system expansion approach, the opportunity cost of diverting the flow of dross from the recycling stream is internalized: the recoverable part (“Al in dross” in Table 1) is modeled as a secondary resource and triggers an environmental impact, while the remaining 38% nonrecoverable fraction is treated as a recovered waste and avoids the impact of its treatment. In Table 1, a summary of the complete inventory and data sources is included. The complete list of equivalences between inventory flows and unit flows in Ecoinvent 3.9.1 is displayed in Supporting Information C.

**Table 1:** Life Cycle Inventory for the Four Scenarios per F.U. of 1 Tonne MG-Si^a^

	Substance	Unit	C	Al. B	Al. WR	Al. NH	Source
Inputs	Si input	tonne	2.68	2.71	2.99	2.22	M
	Woodchips	tonne	0.49	-	-	-	M
	Hard coal	tonne	1.72	-	-	-	M
	CaO	tonne	-	2.74	2.70	0.75	M
	CaCO_3_	tonne	0.08	-	-	-	M
	Electricity	MWh	13.52	4.64	5.14	2.39	M
	Air	tonne	95.00	1.71	1.71	-	M
	Al in dross	tonne	-	0.61	0.61	0.61	M
	Petroleum coke	tonne	-	0.05	0.05	-	M
	Liquid CO_2_	tonne	-	1.57	1.35	-	M
	Nitrogen	tonne	-	4.00	-	-	M
Products and byproducts	MG-Si	tonne	1.00	1.00	1.00	1.00	M
	Microsilica	tonne	0.27	-	-	-	M
	Al_2_O_3_ product	tonne	-	0.70	0.70	-	M
	CaO	tonne	-	-	-	0.91	M
Emissions to air	CO_2_	tonne	5.86	0.54	0.35	0.35	M, L^11^
	CO_2_, biogenic	tonne	0.89	-	-	-	L^54^
	CO	kg	5.22	-	-	-	L^11^
	NOx	kg	11.50	1.15	1.15	1.15	L^36,48^
	Sulfur oxides	kg	15.00	0.38	0.38	0.38	L^36^/M
	CH_4_	kg	1.20	-	-	-	L^37–39^
	Dioxins	μg	3.00	-	-	-	L^40^/E
	PAHs	g	3.00	-	-	-	L^40^/E
	Chlorinated hydrocarbons	ng	37.92	-	-	-	L^11^
	PM < 2.5 μm	g	600.00	0.02	5 × 10^–3^	2 × 10^–4^	L^41^/E^50^
	PM < 10 μm	g	850.00	-	-	-	L^41^
	Aluminum	g	117.31	3.52	3.52	3.52	L^11^
	Antimony	mg	27.05	0.62	0.62	0.62	L^11^
	Arsenic	mg	11.66	0.89	0.89	0.89	L^11^
	Boron	mg	18.40	0.48	0.48	0.48	L^11^
	Barium	mg	5.05	0.25	0.25	0.25	L^11^
	Beryllium	μg	78.51	3.58	3.58	3.58	L^11^
	Calcium	g	169.90	2.39	2.39	2.39	L^11^
	Cadmium	mg	38.96	3.62	3.62	3.62	L^11^
	Cobalt	g	1.87	0.02	0.02	0.02	L^11^
	Chromium	μg	773.67	30.00	30.00	30.00	L^11^
	Copper	g	1.54	0.11	0.11	0.11	L^11^
	Iron	g	61.35	3.22	3.22	3.22	L^11^
	Lead	g	0.36	36.50	35.80	35.90	L^11^/M
	Mercury	μg	18.53	1.51	1.51	1.51	L^11^
	Magnesium	g	0.02	2.63	2.58	2.77	L^11^/M
	Manganese	g	257.22	83.46	83.46	83.46	L^11^
	Molybdenum	mg	166.36	2.00	2.00	2.00	L^11^
	Nickel	μg	402.04	49.39	49.39	49.39	L^11^
	Phosphorus	g	4.51	0.11	0.11	0.11	L^11^
	Potassium	kg	1.65 × 10^–3^	1.49	1.46	0.66	L^11^/M
	Scandium	μg	267.09	-	-	-	L^11^
	Selenium	mg	1.64	0.01	0.01	0.01	L^11^
	Sodium	kg	3.40 × 10^–4^	10.86	10.65	11.33	L^11^/M
	Sodium chloride	kg	-	42.28	41.48	41.52	M
	Strontium	mg	592.91	31.01	31.01	31.01	L^11^
	Thallium	μg	233.96	8.76	8.76	8.76	L^11^
	Tin	mg	65.28	7.66	7.66	7.66	L^11^
	Tungsten	g	1.94	5 × 10^–04^	5 × 10^–04^	5 × 10^–04^	L^11^
	Vanadium	μg	100.34	14.74	14.74	14.74	L^11^
	Zinc	kg	8.41 × 10^–3^	6.10	5.98	5.99	L^11^/M
	Zirconium	μg	262.73	0.68	0.68	0.68	L^11^
	H_2_O vapor	tonne	2.83	0.37	-	-	M
Solid residues	Si-conventional slags	tonne	0.24	-	-	-	M
	Al dross residue	tonne	-	–0.37	–0.37	–0.37	M
	Inert waste	tonne	-	6.20	6.13	1.66	M

a C = Carbothermic; Al = Aluminothermic; B = Baseline; WR = with Recirculation; NH = No-Hydrometallurgy; M = Process Simulation Model; E = Expert Knowledge; L = Literature. Numbers in literature sources refer to the numbers in the reference list.

Systematic differences that can be observed from the inventory are:

- Direct energy consumption decreases substantially in the aluminothermic route, with the aluminothermic reduction occurring at lower temperatures and a more exothermic process. Aluminothermic NH requires the least amount of energy of the process configurations studied as the hydrometallurgical separation is not considered.
- The aluminothermic route can use secondary materials for production. Still, the impurities involved cause a substantial increase in the amount of residues produced. Residues that cannot be recovered undergo further treatment or disposal environmental costs, and the resulting burden is therefore allocated to the waste generating processes with unrecovered waste streams.
- While all systems produce MG-Si, byproducts of the different routes include: microsilica in conventional production (0.27 tonnes), alumina in Aluminothermic B and WR (0.70 tonnes), and CaO in Aluminothermic NH (0.91 tonnes). For alumina and quicklime to be credited as byproducts in the aluminothermic routes, their quality must match that of the products they replace. The process model simulation retrieves alumina at 99.97% purity, with SiO_2_ limited to 0.03% (Figure S3), meeting commercial specifications. To account for the purity of CaO, only the equivalent fraction of the produced slags is replaced, and the rest of slags undergo waste management.
- Emissions to air generally decrease in the aluminothermic route, except for some metal emissions (e.g., K, Na, Pb, Mg, Zn) introduced through the raw materials.

### Impact Assessment

The results of the LCA comparison between the carbothermic and aluminothermic production routes in the different process configurations are displayed in Figure 2.

**Figure 2:**
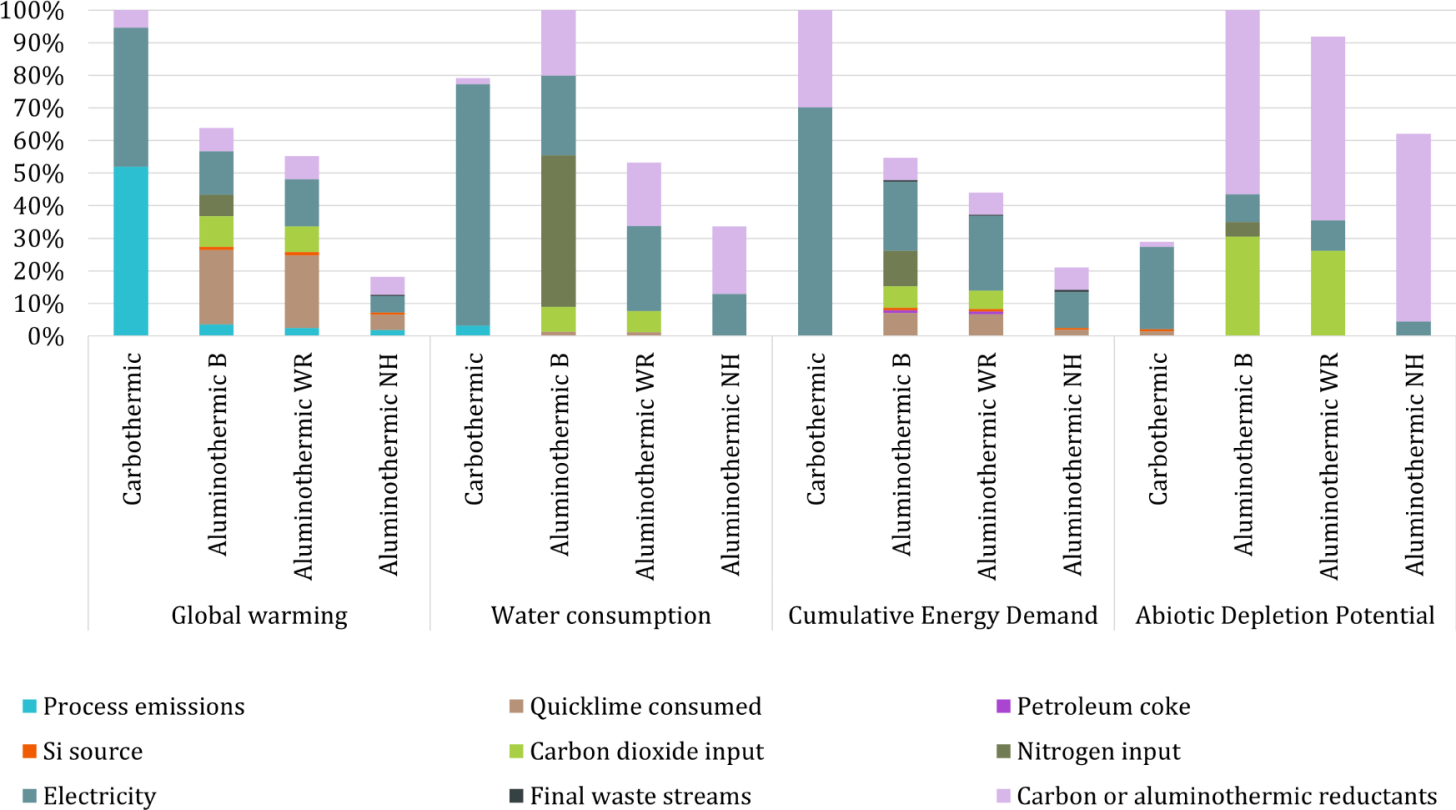
Midpoint evaluation for the selected impact categories following the carbothermic and aluminothermic routes (characterization results, normalized to 100% of the maximum impact per impact category). Cutoff = 0.01.

It is observed that the baseline process for the aluminothermic route outperforms the carbothermic route on global warming and cumulative energy demand impacts. In contrast, the opposite holds for water consumption and abiotic depletion potential, where the conventional route has the lowest impact. The contribution analysis for each impact category is discussed in the following section to understand better where the environmental impacts arise.

#### Contribution Analysis

Figure 2 shows that the impacts of the carbothermic route are mainly derived from the process emissions released in the furnace and the electricity and carbon consumed by this process. As studied in the inventory analysis, greenhouse gas emissions released and electricity consumed are lower in the aluminothermic route. However, there is a substantial contribution to the environmental impact from this route from the quicklime used in the silicon refining, the input of aluminum dross as a reductant, and the carbon dioxide and nitrogen added to refine the slags. The contribution of these inputs to the aluminothermic route is discussed next.

Quicklime is a major contributor to global warming, as its production process releases a large amount of GHG emissions when produced from calcium carbonate (CaCO_3_). The dross applied as feed material is a major contributor to the aluminothermic route’s environmental impact, especially for categories such as abiotic depletion. By system expansion, when dross is not utilized to recover aluminum in the recycling process, this will drive primary aluminum production. Nitrogen and carbon dioxide inputs are also large contributors to global warming due to their carbon- and energy-intensive production.^57,58^ In addition, the input of nitrogen significantly affects water consumption. For these reasons, the overall impact decreases when recirculating the carbonation gas, which contains both CO_2_ and N_2_ in Aluminothermic WR, and more strongly in Aluminothermic NH, where slags are revalorized as CaO replacement in the market.

Characterization results are represented by impact category in Supporting Information D.

#### Sensitivity Analysis

Scenario parameters are modified to simulate different conditions that the market could present when this technology reaches maturity (TRL9), which can reduce the inherent uncertainty of prospective LCAs.^59,60^ The parameters studied are the source of the reductant materials (aluminum and carbon) and a progressive decarbonization of electricity. These parameters are selected because (i) they exhibited the highest contribution to the environmental impact in the contribution analysis and (ii) are expected to experience a significant variability in their environmental impact during scaling up and implementation of the aluminothermic route.

Aluminum raw materials: dross adds to the impact of the aluminothermic route because it is being diverted from the recycling stream, and new aluminum would be produced to compensate for this. The percentage of 62% recovery considered in this assessment varies depending on the type of aluminum residue. Black dross, or the dross generated in secondary smelters with lower Al content than white dross (7–35%),^24^ is generally classified as hazardous, with high recycling and disposal environmental and economic costs, and up to 95% of these residues end up in landfills.^23^ If black dross is used, or when Al is not recycled, reused, or recovered in the technosphere, the impact of diverting it to Si production is nullified. Other conditions might also justify considering the aluminum source free of burden if a large surplus of secondary aluminum is being produced, for instance, due to an overflow of downgraded aluminum residues that might occur as vehicles get discarded,^61^ or through new technologies that could recover secondary materials not currently being utilized, such as fine fractions of zorba that are challenging for hand sorting and end up offloaded for low prices.^62,63^ It is also worth mentioning that even though more aluminum residues might become available, different market conditions, such as developing new alloys, could compete with the aluminothermic route in using these scrap fractions.

Carbon raw materials: the starting point of this analysis is an average reductant feed for the carbothermic route (65% coal and 35% woodchips). While the industry trend is toward substituting carbon reductants with biogenic materials such as charcoal or woodchips to achieve carbon neutrality, their application has other effects regarding land-use changes, different properties of the reductant inside the furnace, increased costs, or competition with other uses for these raw materials.^64^

Electricity: in addition, both the carbothermic and aluminothermic route will likely see an increase in decarbonized energy sources in the electricity mix. Together with replacing carbon anodes, this might lead to zero-carbon emission aluminum smelters.^65^

The variation of these parameters is introduced in Figure 3.

**Figure 3:**
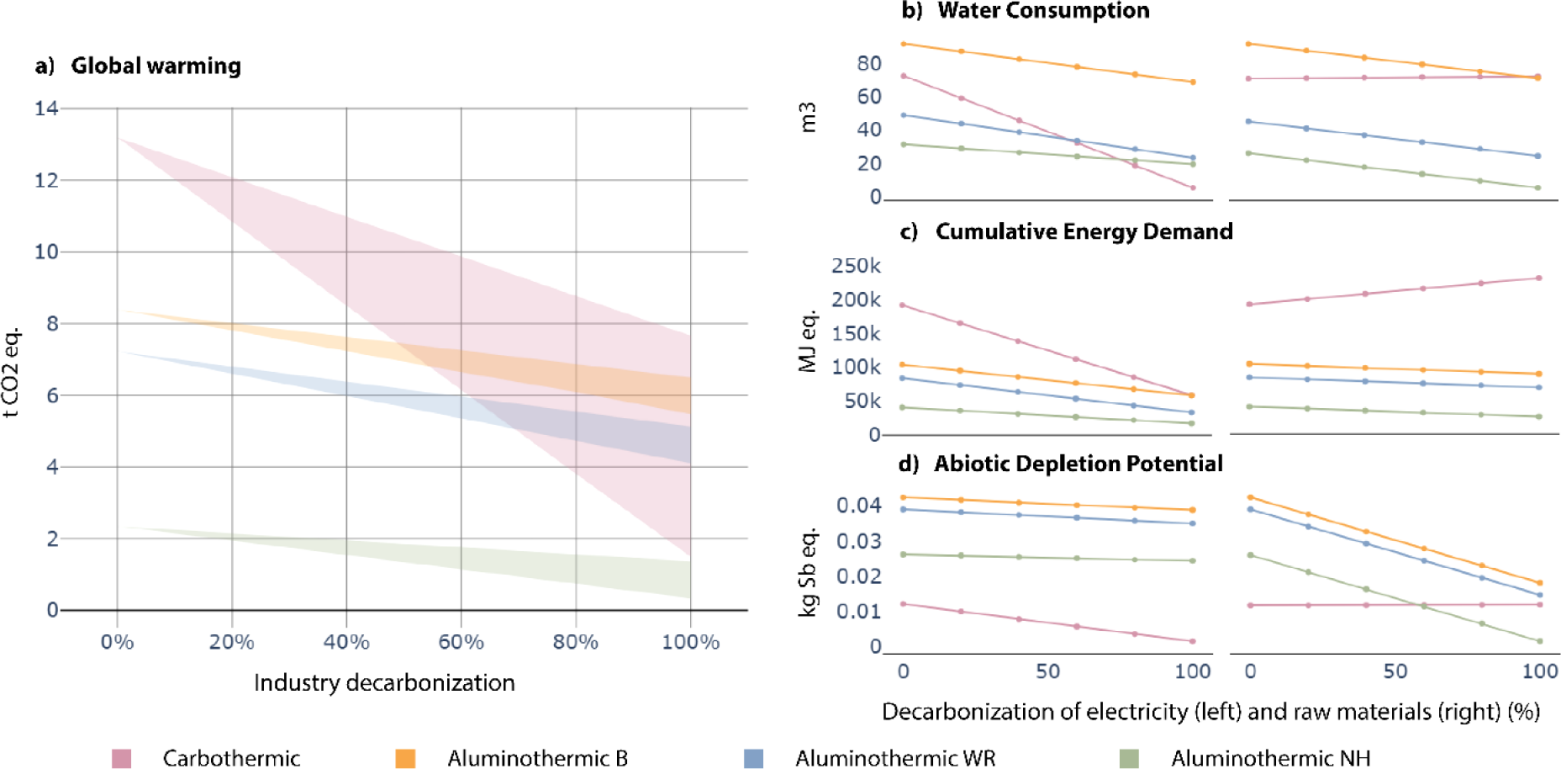
Industry decarbonization effect on impact for the different process configurations. a) The combined effect of both background and foreground changes on global warming. The top edge of each color shading reflects the impact of a progressively decarbonized electricity mix. The bottom edge represents the total impact when substituting fossil-based reductants with biocarbon in the carbothermic route, and using free-of-burden drosses in the aluminothermic route, alongside a decarbonized electricity mix. b–d) Effect of the decarbonization of electricity (left) and raw materials (right) on water consumption, cumulative energy demand, and abiotic depletion potential.

It is observed that both routes are highly sensitive to the decarbonization of industry, especially the carbothermic route related to higher electricity consumption and reliance on fossil carbon. An increase in the carbon content in the feed mix can significantly decrease the impact of global warming on the carbothermic route but at the expense of other impacts. To study this effect, Figure 3b–d displays separately electricity and raw material decarbonization for water consumption, cumulative energy demand and abiotic depletion impacts. The substitution of fossil materials by biocarbon increases the impact of categories such as water consumption and cumulative energy demand, partially because a lower fixed carbon content in woodchips requires a larger amount of these to compensate for the substituted fossil carbon.

Because of the different availability of raw materials and electricity mixes, the geographical location where the carbothermic and aluminothermic routes are to be implemented is crucial in the environmental assessment.

Aluminothermic NH appears to be the least impactful across most impact categories and implementation conditions. However, this route does not produce alumina but a calcium-aluminate slag, so the decision to implement one or the other will rely on the specific material demand.

#### Uncertainty Evaluation

We apply mass and energy-balanced process simulation as a basis for the LCI. Foreground data uncertainty is minimized through process simulation when plant data is not available.^10^ Some parameters were taken from literature and best estimates, which could be older and less suited to our model. To deal with this uncertainty, some impact categories had to be excluded from the analysis (i.e., particulate materials, toxicity impacts). A pedigree matrix^66^ is displayed in Supporting Information E for the evaluation of uncertainty in the inventory.

Fixed infrastructure is excluded from the inventory. Because the aluminothermic route is a multifunctional process providing both silicon and a waste management service, it displaces part of the conventional infrastructure under the system expansion approach, where the allocation of the avoided burden depends on future market conditions inherently uncertain. Moreover, because the impact of capital goods is amortized over decades of output, their share in total impacts is unlikely to affect the comparison results.

Another simplification made is in the energy recovered. In conventional production, a heat exchanger can be placed after the furnace to recover around 20% of the energy in the off-gas.^67^ In the aluminothermic route, this efficiency is not known. Therefore, it was decided to exclude energy recovery from both systems, which is not expected to influence the overall conclusions of this study greatly but should be considered when more data is available.

The model’s connection between foreground and background data is also associated with uncertainty, as unit processes not defined in our background database were excluded. For instance, this study uses silicon sculls instead of primary silicon as a source of Si. Silicon sculls could also be used as a secondary raw material for silicomanganese production.^67^ However, no quantitative data was found on the use of sculls by the silicomanganese industry, and these were assumed to be equivalent to primary materials after upscaling by Si content. Silica sand stands for less than 5% of the impact for most impact categories and scenario configurations and is not considered a significant source of uncertainty for the results.

### Final Remarks

The increasing demand for electronics and solar energy applications in the coming years makes it paramount to reduce the environmental impact of silicon production in order to achieve a sustainable society. The prospective LCA applied in this study indicates that aluminothermic MG-Si can reduce GHG emissions by 36–80% compared to the conventional carbothermic route, depending on the analyzed process configuration. However, other impacts, such as water consumption and abiotic depletion potential, might increase due to the input of quicklime, aluminum dross, carbon dioxide and nitrogen associated with this route.

Through prospective LCA we were able to detect early challenges of the application of the aluminothermic production of silicon, and study some options to reduce the hotspots of environmental impact, including recirculating the carbonation gases, reprocessing the byproduct slags, and using free-of-burden Al, all of which can decrease the environmental impact across impact categories relative to the baseline aluminothermic route. A sensitivity analysis is then performed to account for future market conditions and availability of raw materials, including the use of biocarbon and decarbonized electricity mixes. A future green electricity mix favors both routes, as the carbothermic route consumes more electricity, and the aluminothermic route requires aluminum, which is an energy-intensive material in production. The prioritization of technology will therefore depend on local market conditions and the availability of raw materials, which are likely to vary by region; hence, regional assessments are recommended.

Due to the prospective nature of this study, scaling up from pilot to industrial practice introduces unavoidable uncertainty. Future work should prioritize gathering plant-scale data to account for missing impact categories and end point trade-offs that are not covered in this study. Beyond the environmental dimension, economic and social aspects should also be examined, as well as end-user acceptance as the product approaches commercialization. Furthermore, process improvements, such as integrating calcium looping into the aluminothermic route, can be explored to enhance competitiveness and environmental performance.

The sustainability paradox of critical raw materials, which are both essential for the sustainability transition but also create environmental externalities that oppose their role in decarbonization, should be addressed without falling into techno-optimiztic solutions. Prospective Life Cycle Assessment in support of sustainable metallurgy has proven useful toward developing the silicon production process in a sustainable direction, thereby strengthening the importance of the assessment developed as a potential example of industrial symbiosis between raw material industries.

## Supplementary Material



## Notes

HSC process modeling flowsheets, LCI literature data, ecoinvent links, contribution analyses per impact category, and pedigree evaluation (PDF)
