# Supplementary material for: Carbon-Neutral Silicon via Aluminothermic Reduction? Exploring Industrial Symbiosis through Life Cycle Assessment
Source: ACS Sustain Chem Eng. 2025 Sep 2;13(36):14893–902. doi: 10.1021/acssuschemeng.5c04666 (PMC12442225; doi:10.1021/acssuschemeng.5c04666)
Supplement: Supplementary file 1 [file sc5c04666_si_001.pdf]

Supporting Information for:

Carbon-neutral silicon via aluminothermic reduction? Exploring industrial symbiosis through Life Cycle Assessment

ACS Sustainable Chemistry & Engineering

Elisa Pastor-Vallés[a*] · Alejandro Abadías Llamas[b] · Johan B. Pettersen[a]

[a] Industrial Ecology Programme, Department of Energy and Process Engineering, NTNU Norwegian University of Science and Technology. Kolbjørn Hejes vei 1B, 7034 Trondheim, Norway.

[b] Nordenham Metall GmbH, Nordenham 26954, Germany.

*Corresponding author: elisa.p.valles@ntnu.no

This Supporting Information contains:

A HSC flowsheets (pages S2-S5, Figures S1-S4).

B Other literature data used in the LCI (page S6, Tables S1-S2).

C Inventory links to Ecoinvent (pages S7-S8, Table S3).

D Contribution analysis for each impact category (pages S9-S10, Figures S5-S8).

E Pedigree analysis (page S11, Table S4).

References to the Supporting Information (page S12).

A HSC flowsheets

Thermodynamic process simulation models in HSC Chemistry are developed to represent the metallurgical processes for the aluminothermic and carbothermic routes through unit operations. The aluminothermic route is divided in three different flowsheets (Fig. S1-S3).

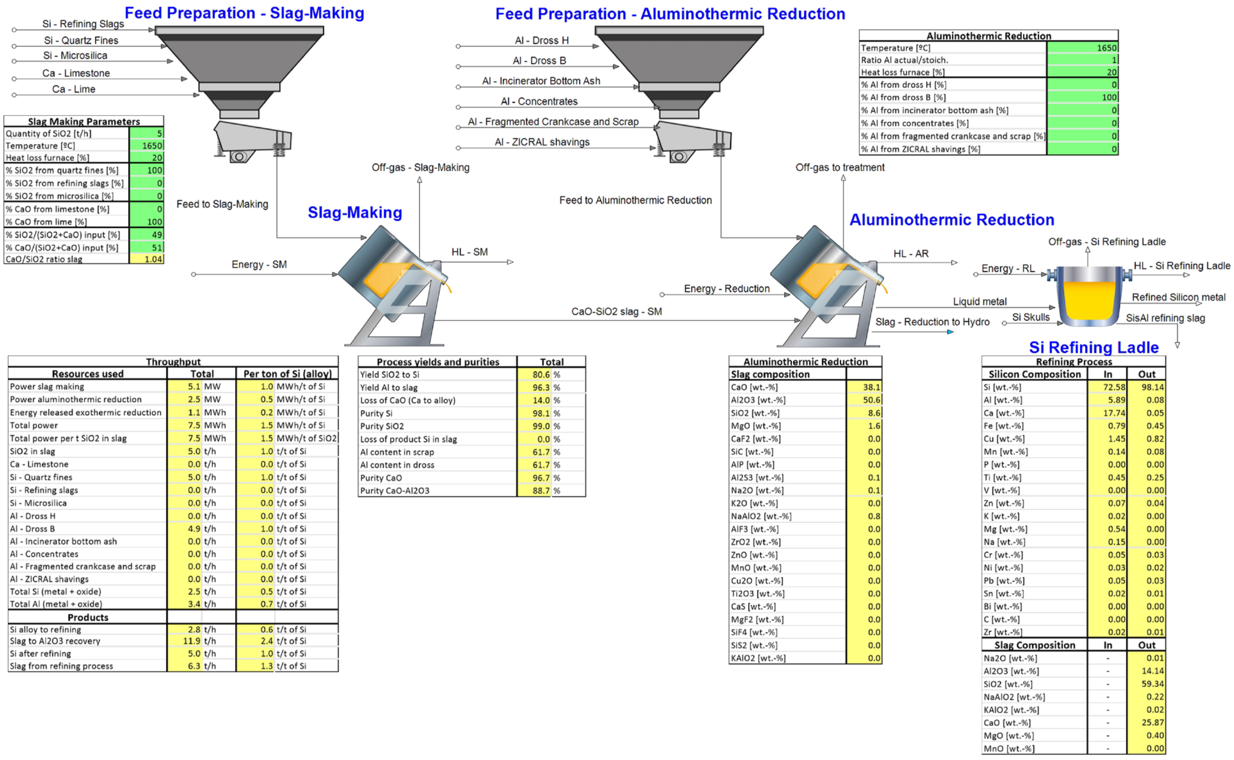

Fig. S1: Simulation model for the aluminothermic route: slag-making, aluminothermic reduction and Si refining steps.

Fig. S1 contains the complete aluminothermic reduction route, including the slag-making, aluminothermic reduction and silicon refining processes. The data on the operating parameters of these processes, e.g. CaO/SiO_2 ratio, aluminum reductant required and temperature, were defined based on the experiments' results. Furthermore, the distribution ratios between different phases for the aluminothermic reduction and silicon refining processes were obtained by performing chemical equilibrium calculations with FactSage for the initial raw material compositions and operating parameters used within the SisAl project. This model was validated with experimental data and internal project meetings with pilot and industrial partners. The products of this flowsheet are (i)

metallurgical grade silicon, (ii) a $CaO-Al_2O_3$ slag that is treated to extract and recover alumina, and (iii) a $CaO-SiO_2-Al_2O_3$ slag after refining.

The second flowsheet, shown in Fig. S2, prepares the slag from reduction to be leached in the hydrometallurgical leaching. This is done by changing the composition of the slag so that the most leachable phases precipitate when it is cooled down. The SiO_2 to Al_2O_3 and Al_2O_3 to CaO ratios come from the consortium's internal data. The precipitated phases were calculated with FactSage. After preparation, the slag is sent to alumina extraction.

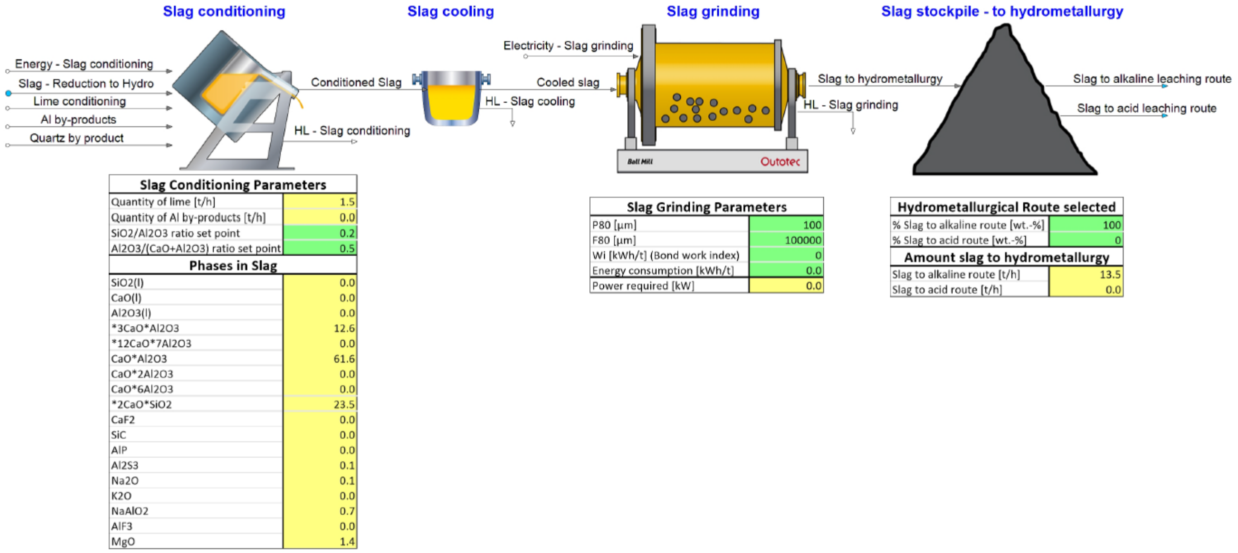

Fig. S2: Flowsheet for the slag preparation for alumina extraction and recovery in the hydrometallurgical process.

In Fig. S3 the leaching, removal, precipitation and calcination stages from extracting and recovering alumina from the slag are developed by using an alkaline leaching route. The operating and leaching parameters were obtained from the experimental work performed within the SisAl project. This flowsheet was validated with the partners conducting the leaching experiments in the project.

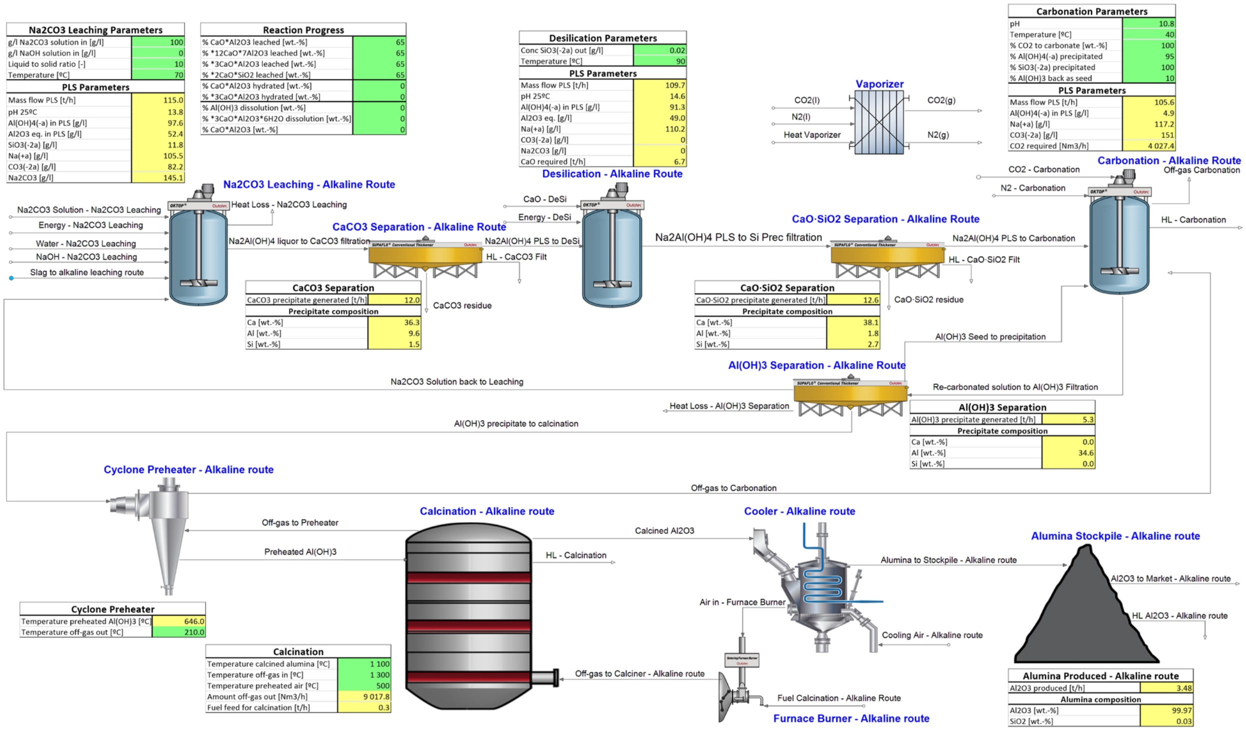

Fig. S3: Flowsheet for the extraction and recovery of alumina through alkaline leaching, precipitation and calcination.

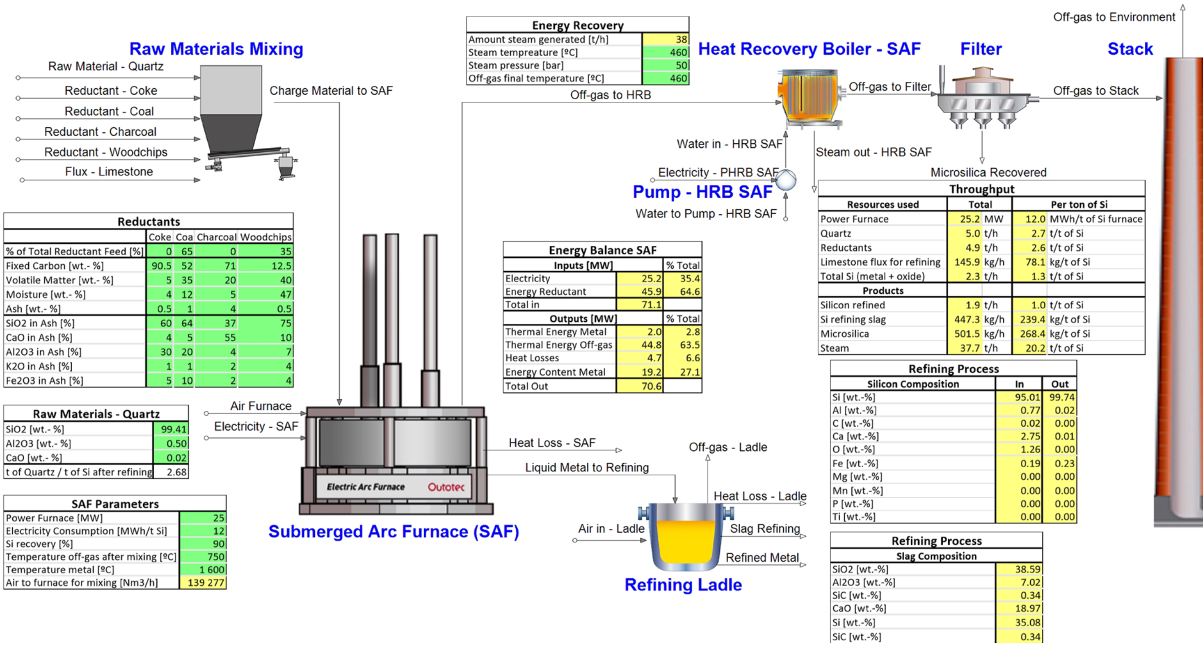

Fig. S4: Flowsheet for the production of silicon through the carbothermic route using a Submerged Arc Furnace (SAF).

The carbothermic route for producing silicon was also simulated so that a dataset for the comparison with the SisAl process is obtained, shown in Fig. S4. The data for model development and validation was obtained from industrial partners of the SisAl process and literature [1].

B Other literature data used in the LCI

Table S1: Emissions factors for the carbothermic route.

Substance	Data	Source	Comments
NOx	10-13 kg/t Si	[2]	Average selected
SO_2	10-18 kg/t Si		
CH_4	1,2 kg/t Si	[3–5]	Assumed to be the same as in ferroalloys production
Dioxins	3 µg/t Si	[6]	
PAH	3 g/t Si		
PM2.5 and PM10	600 g/t Si and 850 g/t Si, respectively	[7]	

Table S2: Carbon content of reductants for biogenic emissions.

Reductant	Total carbon	Source
Woodchips	49,60%	[8]
Charcoal	82,15%	

C Inventory links to Ecoinvent

Table S3: Correspondence between inventory unit flows and Ecoinvent 3.9.1

	Substance	Ecoinvent name
Inputs	Si input	Silica sand (GLO) | market for | APOS
	Woodchips	Wood chips, wet, measured as dry mass (Europe without Switzerland) | market for | APOS
	Hard coal	Hard coal (Europe, without Russia and Turkey) | market for hard coal | APOS
	CaO	Quicklime, in pieces, loose (RoW) | market for quicklime, in pieces, loose | APOS
	$CaCO_3$	Calcium carbonate, precipitated (RER) | market for calcium carbonate, precipitated | APOS
	Electricity	Electricity, medium voltage (ENTSO-E) | market group for | APOS
	Al in dross	Aluminum, wrought alloy (RER) | treatment of aluminum scrap, post-consumer, prepared for recycling, at remelter | APOS
	Petroleum coke	Petroleum coke (GLO) | market for | APOS
	Liquid CO_2	Carbon dioxide, liquid (RER) | market for | APOS
	Nitrogen	Nitrogen, liquid (RER) | market for | APOS
Products and by-products	MG-Si	Functional Unit
	Microsilica	Silica sand (GLO) | market for | APOS

	Al_2O_3 product	Aluminum oxide, metallurgical (IAI Area, EU27 and EFTA) | aluminum oxide production | APOS
	CaO for cement	Quicklime, in pieces, loose (RoW) | market for quicklime, in pieces, loose | APOS
Solid residues	Si-conventional slags	Slag from metallurgical grade silicon production (GLO) | market for | APOS
	Al dross residue	Dross from Al electrolysis (GLO) | market for | APOS
	Inert waste	Inert waste (Europe without Switzerland) | market for inert waste | APOS

D Contribution analysis for each impact category

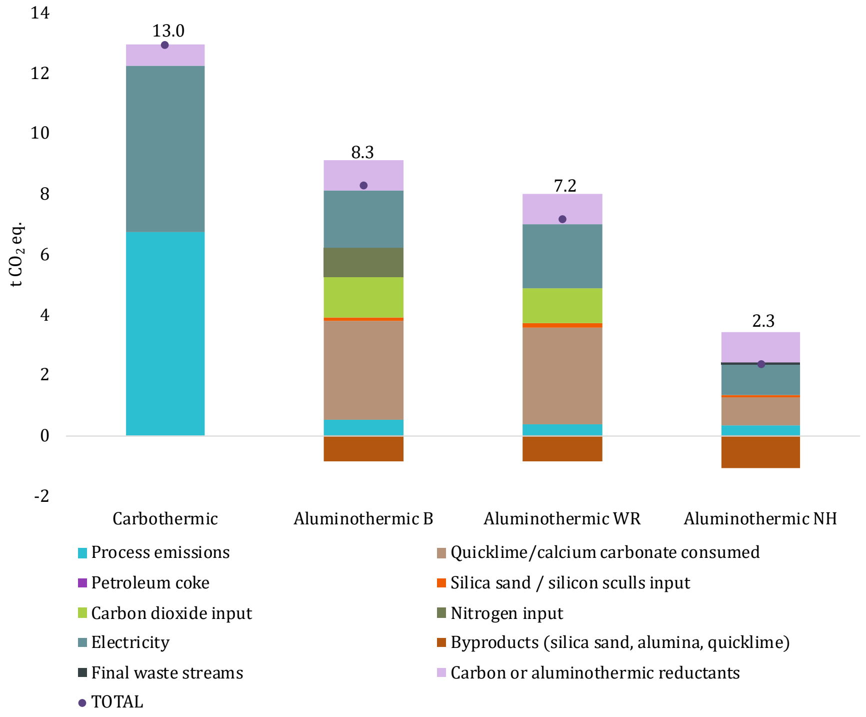

Fig. S5: Characterization results for global warming. Cutoff = 0.01.

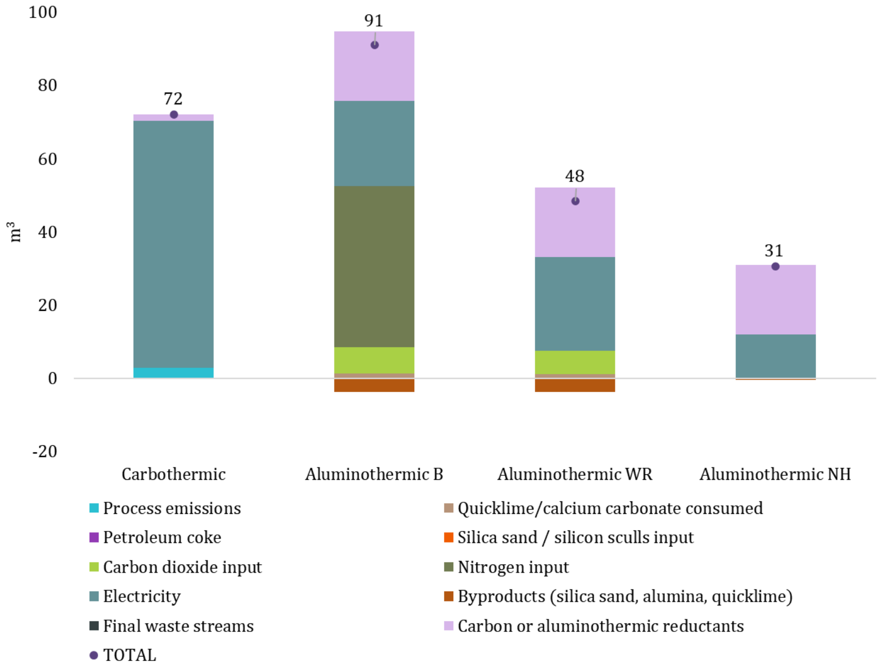

Fig. S6: Characterization results for water consumption. Cutoff = 0.01.

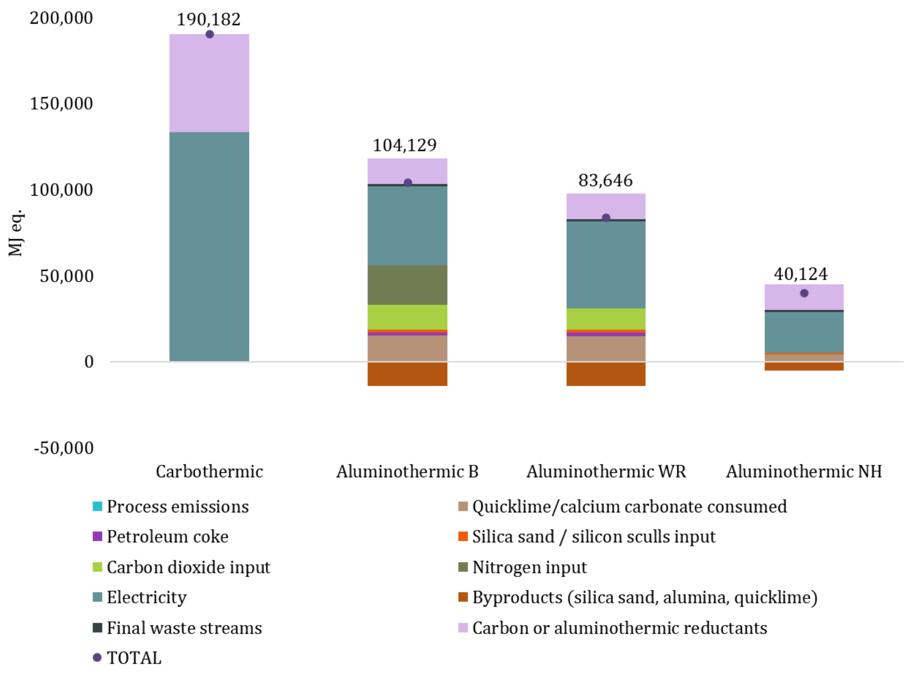

Fig. S7: Characterization results for cumulative energy demand. Cutoff = 0.01.

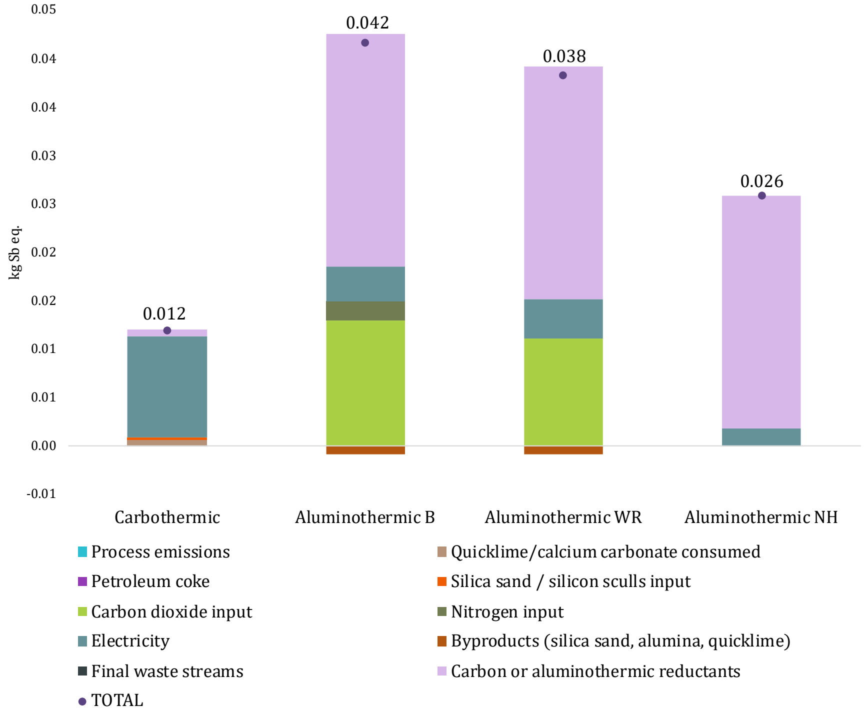

Fig. S8: Characterization results for abiotic depletion impact. Cutoff = 0.01.

E Pedrigree analysis

Table S4: Pedigree Inventory Analysis (based on Ciroth et al., 2016)[9]

Flow	Reliability	Completeness	Temporal correlation	Geographical correlation	Further technological correlation
Inputs	1	2	1	1	1
Products	1	2	1	1	1
CO_2	2	2	1	1	1
Biogenic CO_2	1	3	2	2	2
Nox and SOx	3	2	3	2	2
CH_4	1	1	3	2	2
Dioxins and PAH	2	1	3	2	2
PM	2	1	2	1	2
Other emissions to air	2	2	1	2	2
Solid residues	1	2	1	1	1

REFERENCES

(1) Schei, A.; Tuset, J. K.; Tveit, H. *Production of High Silicon Alloys*; Tapir Academic Press: Trondheim, 1998.
(2) Joint Research Centre. *Best Available Techniques (BAT) Reference Document for the Non-Ferrous Metals Industries: Industrial Emissions Directive 2010/75/EU (Integrated Pollution Prevention and Control)*; Publications Office of the European Union: Luxembourg, 2017. https://data.europa.eu/doi/10.2760/8224 (accessed 2022-07-05).
(3) IPCC. *2019 Refinement to the 2006 IPCC Guidelines for National Greenhouse Gas Inventories*; Volume 3, Chapter 4; 2019. https://www.ipcc-nggip.iges.or.jp/public/2019rf/vol3.html (accessed 2025-07-08).
(4) IPCC. *2006 IPCC Guidelines for National Greenhouse Gas Inventories*; Volume 3, Chapter 4; 2006. https://www.ipcc-nggip.iges.or.jp/public/2006gl/vol3.html (accessed 2025-07-08).
(5) Lindstad, T.; Olsen, S. E.; Tranell, G. Greenhouse Gas Emissions from Ferroalloy Production. In *INFACON XI. Official Proceedings*; Laxmi Publications: New Delhi, India, 2007; pp 457–466.
(6) Sandmo, T. *The Norwegian Emission Inventory 2016. Documentation of Methodologies for Estimating Emissions of Greenhouse Gases and Long-Range Transboundary Air Pollutants*; 2016/22; Statistics Norway: Oslo–Kongsvinger, 2016. https://www.ssb.no/en/natur-og-miljo/artikler-og-publikasjoner/the-norwegian-emission-inventory-2016 (accessed 2024-06-07).
(7) European Environment Agency. *EMEP/EEA Air Pollutant Emission Inventory Guidebook*; Publications Office of the European Union: Luxembourg, 2019.
(8) *ECN Phyllis Classification Database*; TNO, Series Ed.; 2021.
(9) Ciroth, A.; Muller, S.; Weidema, B.; Lesage, P. Empirically Based Uncertainty Factors for the Pedigree Matrix in Ecoinvent. *Int J Life Cycle Assess* **2016**, *21* (9), 1338–1348. https://doi.org/10.1007/s11367-013-0670-5.
